# The Role of the Oral Microbiome in Modulating Therapeutic Outcomes in Lung Cancer: Key Commensals and Clinical Implications

**DOI:** 10.3390/cancers18040591

**Published:** 2026-02-11

**Authors:** Tiara Hening Pertiwi, Ratoe Suraya, Masaya Akashi, Tatsuya Nagano

**Affiliations:** 1 Department of Oral and Maxillofacial Surgery, Graduate School of Medicine, Kobe University, Kobe 650-0017, Japan; drg.tiarahp@gmail.com (T.H.P.); akashim@med.kobe-u.ac.jp (M.A.); 2 Division of Respiratory Medicine, Department of Internal Medicine, Graduate School of Medicine, Kobe University, Kobe 650-0017, Japan; dr.ratoesuraya@yahoo.com

**Keywords:** lung cancer, oral microbiome, treatment outcomes, chemotherapy, radiotherapy, immunotherapy

## Abstract

Lung cancer remains one of the deadliest forms of cancer worldwide; thus, there is an urgent need to continually devise new strategies to effectively treat this condition. In this review, we will discuss one of the previously less studied but important aspects of the oral microbiome in both accelerating the development of lung cancer and modulating the efficacy of its treatment modalities. Herein, following an exhaustive search of available databases, we summarize the current knowledge on the association between oral microbiota and lung cancer, focusing on its impact on the efficacy and complications of widely used lung cancer treatments, including surgery, radiotherapy, chemotherapy, and immunotherapy. We also discuss the evidence supporting the use of oral microbiome-targeting interventions in improving outcomes in lung cancer, both preclinically and clinically, and conclude that the full potential of modulating oral dysbiosis in lung cancer has yet to be realized, requiring broader and larger-scale studies in the future. We hope that this review will highlight the importance of an often-forgotten aspect of lung cancer treatment optimization.

## Simple Summary

Due to a variety of underlying factors, the oral microbiome is a sometimes disregarded yet unquestionably significant factor that can change the course and overall outcome of lung cancer therapy. In recent years, dysbiosis in the oral microbiome has been correlated not only with the occurrence of lung cancer but also with its treatment outcome. Accordingly, several intervention strategies, such as probiotics, antimicrobial peptides, and oral hygiene care, have been developed to combat oral dysbiosis in lung cancer conditions with varying levels of success; more research is necessary to validate these strategies as a supplement to the available lung cancer treatments and maximize their effectiveness.

## 1. Introduction

Cancer remains one of the deadliest conditions worldwide, with lung cancer representing one of the most prevalent types [1]. Although the advent of novel therapeutic options such as targeted therapies and immunotherapies has been instrumental in improving the outcomes of lung cancer patients, the prognosis of this condition remains poor [2]. To address this challenge, new treatment approaches and optimization strategies must be continuously developed. One of the recently linked, understudied aspects that determines the success of lung cancer therapy is the microbiota of the respiratory tract, specifically the oral microbiome. Human microbiomes are crucial for preserving host homeostasis [3]. Specifically, the oral microbiome—the second most diverse microbiota in the human body with over 800 species and 20 million nonredundant genes—is essential for establishing connections with the external world through the respiratory and digestive tracts [4]. Importantly, as highlighted in subsequent sections, lung cancer is one of the systemic diseases that can be induced by oral microbiome dysbiosis, with oral dysbiosis potentially also leading to altered outcomes in lung cancer patients. Indeed, one of the often overlooked yet significant components of the oral microbiota influences the effectiveness of many primary lung cancer treatment methods, which will be addressed in this review. Herein, following a search (conducted on October 2025) of the literature from the last ten years from available databases (PubMed, Web of Science, Scopus, and Cochrane Library) using multiple different search term such as “oral microbiome”, “oral dysbiosis”, “lung cancer”, “non-small cell lung cancer”, “small cell lung cancer”, “food therapy”, “oral care”, “probiotic”, “antimicrobial peptides”, “chemotherapy”, “immunotherapy”, “radiotherapy”, “lung cancer development”, and “lung cancer therapy outcome”, we methodically reviewed the current understanding of the relationship between the oral microbiota and lung cancer, with a focus on how it influences the efficacy and toxicity of commonly used lung cancer treatments, such as radiation, chemotherapy, immunotherapies, and surgical resection.

## 2. The Current Landscape of Lung Cancer and Its Therapeutic Options

Cancer represents a major health burden that significantly contributes to overall morbidity and mortality. Among the various types of cancer, such as gastric, colon, breast, or cervical cancer, lung cancer has historically ranked as one of the most prevalent types of cancer worldwide [1]. Lung cancer, together with tracheal and bronchial cancer, accounted for the greatest number of cancer cases worldwide in 2022 across all five continents. Based on data from the World Cancer Research Fund Organization, 2,480,675 cases of lung cancer were recorded worldwide in 2022 [5]. The geographic burden is extremely uneven; by continent, Asia accounts for roughly two-thirds of all cases and deaths worldwide [6]. The incidence of lung cancer is predicted to rise by more than 60% from 2022 to 2050 [7]. Of particular note is that non-small cell lung cancer (NSCLC), which accounts for 85% of all instances of lung cancer worldwide, is characterized by a 5-year survival rate of 18.6% for newly diagnosed patients [8]. In light of this poor prognosis, it is imperative to understand all aspects that could affect lung cancer treatment to ensure the best possible outcome for patients. Regarding lung cancer therapy, while comprehensive therapeutic options differ among each cancer subtype, we are now approaching a precision medicine approach, which generally includes surgery, chemotherapy, radiotherapy, targeted therapy, and immunotherapy. Up until the 1970s, despite surgery and more effective supportive treatment, the median overall survival for metastatic lung cancer stood at a mere two to four months before the advent of chemotherapy [9]. In the 1970s, lung cancer was managed using early-generation chemotherapeutic drugs, including doxorubicin and methotrexate, with minimal clinical benefits, however [9]. Only with the development and introduction of platinum and next-generation chemotherapeutic drugs, such as taxanes, vinorelbine, and gemcitabine, in the 1980s and 1990s was there a discernible improvement in lung cancer patients’ survival through several landmark clinical trials, and chemotherapy started to be recommended as the first-line treatment in NSCLC in combination with platinum [10]. Treatment was improved further with the introduction of pemetrexed, which provided proven efficacy. In the mid-2000s and early 2010s, respectively, anti-angiogenesis medications, namely, bevacizumab, and novel drug delivery methods such as nanoparticle albumin-bound paclitaxel (nab-paclitaxel) demonstrated clinical benefit in terms of efficacy and tolerability and were approved as new, legitimate agents for the treatment of lung cancer [11].

In more recent years, chemotherapy-based combinational strategies employing tyrosine kinase inhibitor (TKI)-based targeted therapies or immune checkpoint inhibitors (ICIs) have demonstrated a survival advantage over chemotherapy alone [12,13]. Through a variety of signaling pathways, tyrosine kinases are enzymes that control important cellular functions, such as proliferation, apoptosis, differentiation, and survival. TKIs are therefore molecularly targeted treatments that can stop the dysregulated signaling pathways that cause cancer cells to proliferate abnormally [14]. The advent of TKIs has particularly revolutionized NSCLC treatment, especially in those with EGFR-mutated cancers, wherein it significantly improves survival [15]. TKIs are currently used in metastatic, adjuvant (after surgery), and induction (before surgery) settings, showing promise in shrinking tumors and improving surgical eligibility [15]. Even more recent is the usage of ICIs, which inhibit immune checkpoints by targeting PD-1/PD-L1 or CTLA-4 [16]. The approval and clinical usage of ICIs in recent years have improved the standard of care for advanced NSCLC by reactivating T cells to target tumors, significantly improving survival, particularly in patients with high PD-L1 expression [16]. While highly effective for many, not all patients respond positively to this therapeutic option. Still, despite the numerous novel options for treating lung cancer, there remains considerable room for improvement in the outcomes of lung cancer therapy because these therapies are still strongly influenced by multiple other factors. Among the influencing factors, the authors of multiple studies have linked various biotic factors, including microbiomes, with the growth, progression, and response to treatment in multiple types of cancer. Changes in organ microbiota, not only at the cancer site but also in connected or surrounding organs, have been reported to be concurrently found in cancers at an advanced stage. As will be discussed in subsequent sections, the oral microbiome is one of the factors that modulates both the development and treatment outcomes of lung cancer.

## 3. The Physiology of the Oral Microbiome

It is crucial to first understand how the oral microbiome is physiologically conditioned before examining its association with lung cancer. More than 800 microbial species, including bacteria, fungi, viruses, protozoa, and archaea, constitute the complex ecosystem known as the oral microbiome [17]. Many oral surfaces, including the tongue, teeth, mucosa, gingiva, tonsils, and saliva, harbor these microorganisms, with examples shown in Table 1 [4,17,18]. The composition of the microorganisms, their physiology, and the overall ecological balance are heavily influenced by a myriad of host and environmental factors, such as pH, host immunological interactions, oxygen gradients, and nutritional availability [4,17]. Beginning with the early colonization of oral surfaces by pioneer species such as *Streptococcus* and *Actinomyces*, which initiate biofilm formation and define subsequent microbial succession, these communities maintain physiological homeostasis through coordinated microbial functions [19].

While commensal streptococci contribute to microbial stability by generating hydrogen peroxide, which inhibits opportunistic pathogens, cooperative interactions within these biofilms maintain metabolic balance [20]. By buffering pH, producing antibacterial peptides, and supplying nutrients that control microbial composition and avoid over-acidification, saliva also serves a crucial physiological role [21]. A healthy host–microbe interaction is made possible at the host level by ongoing low-grade immune monitoring, which encourages tolerance rather than inflammation. When combined, these processes create a robust, mutually beneficial ecosystem that protects oral tissue integrity and guards against diseases linked to dysbiosis [22].

The oral microbiome’s stability and structure are heavily impacted by behavioral and external variables that alter its ecological balance, in addition to internal physiological processes. By inducing anaerobic conditions, weakening human immunological and salivary responses, and increasing microbial pathogenicity, smoking significantly changes the oral microbiome, disturbing microbial homeostasis and promoting the colonization of pathogenic species. These alterations are accompanied by compositional changes, such as an increase in anaerobe-associated taxa and a decrease in *Proteobacteria*, which together increase vulnerability to oral infections and illness [4].

Microbial communities are further shaped by oral hygiene practices; insufficient plaque control and infrequent brushing cause site-specific changes on oral surfaces, which interfere with the normal structuring of biofilms [4]. Furthermore, by decreasing community diversity and eradicating protective commensals, antibiotic exposure can disrupt oral microbial homeostasis, potentially allowing opportunistic microorganisms to proliferate during recolonization [4]. When taken as a whole, these environmental and lifestyle variables interact with host physiology to affect oral microbial resilience and composition. These factors are crucial in determining whether the oral microbiome stays in a state of homeostasis or moves toward dysbiosis.

## 4. Oral Dysbiosis in Lung Cancer Patients

Although the oral microbiota may initially appear unrelated to lung cancer, recent paradigms redefining the human respiratory tract highlight the importance of the oral cavity, which is now deemed a crucial gateway shared by both the digestive and respiratory systems, underscoring its central role in regulating overall health [23]. Thus, any imbalance in the oral microbiota environment could lead to a chain reaction that can cause alterations in multiple aspects of lung cancer. Regarding lung cancer patients or those at risk of developing lung cancer, oral dysbiosis may occur mainly due to smoking. Smoking, or use of tobacco in general, has been linked to alterations in the oral microbiome due to chronic alteration of oxygen and pH levels, changes in the immune system due to the chronic insult by chemicals, and suppressed saliva production, among others [24]. In addition, various other factors, such as a high-sugar diet, poor routine oral care, excessive alcohol consumption, and the use of antibiotics, can contribute to oral dysbiosis [25].

With a diverse array of bacterial, viral, and fungal communities, the oral cavity is one of the human body’s most complex microbial ecosystems, and mounting evidence suggests that oral microbiota dysbiosis may aid in accelerating lung carcinogenesis [26]. First, the vital function that microbial communities play in preserving the integrity of the epithelial barrier and immunological homeostasis is important in maintaining immune function. The commensal microbiota actively influences the formation and function of cytokine- and chemokine-activated dendritic cells (DCs), facilitating rapid host defense [27]. Furthermore, increased presence of *Streptococcus sanguinis*-related bacteria can cause damage at the oral mucosal interface by activating Langerhans cells and causing cross-reactive autoimmunity that targets homologs of epithelial heat shock proteins [28]. This mechanism is responsible for the epithelial damage caused by cytotoxic T lymphocytes, which is a feature of recurrent aphthous ulceration [29]. Microbial changes are stage-specific, with advanced disease exhibiting enrichment of certain taxa (such as *Legionella*), accompanied by alterations in the signaling pathways during advanced, stage IV lung cancer that correspond with the alteration in microbiota and promote further chronic inflammation and altered metabolism [28].

Another aspect by which oral dysbiosis may alter pulmonary microbial homeostasis and contribute to lung carcinogenesis is direct translocation to the lungs through hematogenous dissemination or micro-aspiration. Chronic inflammation induced by periodontal infections such as *Fusobacterium nucleatum* or *Aggregatibacter actinomycetemcomitans* may mediate this process [30]. This hypothesis is confirmed by recent research demonstrating notable changes in bacterial burdens in cancer cells compared to surrounding cells [31]. For example, NSCLC tumors possess lower α- and β-diversity and a notable depletion in *Fusobacterium* and *Streptococcus* than nearby healthy tissue, with a corresponding enrichment of potentially harmful microbiota such as *Aeromonas*, *Sphingomonas*, or CW040, some of which are linked with an increased risk of lung cancer development through various means, such as the production of microbial metabolites that are potentially pathogenic [32,33,34]. Additionally, patients with lung cancer also possess different salivary microbiome profiles, even among the non-smoking population [35]. This finding suggests that salivary microbiota can be an informative source for identifying non-invasive lung cancer biomarkers and may reveal correlations between the salivary microbiome and immunocytochemistry markers used in clinical diagnostics. To summarize, the two main contributions of oral dysbiosis in accelerating lung cancer include alteration in immune cell response and inflammation processes, as well as pro-carcinogenic direct translocation into the lungs, where it helps promote carcinogenesis.

Regarding lung cancer therapies, one of the challenges that emerges with oral dysbiosis is related to the occurrence of post-surgery comorbidities and complications. It is widely established that multiple adverse effects related to immunomodulating therapies, starting from oral mucositis or infections up to pneumonia, could severely hamper the immediate health status of the patient [36,37]. These factors can lead to reduced responsiveness to therapy among patients with oral dysbiosis. The authors of one study highlighted how patients with lung cancer possess unique and dynamic oral microbial fingerprints that are strongly correlated with survival rates, treatment response, and disease state. While non-responder-associated taxa (*Porphyromonas endodontalis*) and responder-enriched taxa (*Rothia aeria* and *Prevotella salivae*) predict radiation efficacy and survival, the presence or absence of *Streptococcus* distinguishes patients from healthy individuals [38].

In addition, cancer therapy is also linked with several associated comorbidities (e.g., neutropenia, anemia, and impaired wound healing) that can complicate periodontal maintenance and treatment and even cause additional comorbidities that increase the burden for lung cancer patients. In a recent study, Tsuji et al. completed a prospective investigation of dental intervention for patients with hematopoietic malignancy, reporting that patients who completed the partial pre-cancer therapy protocol exhibited a significantly lower incidence of systemic and dental complications compared to patients who did not complete any pre-cancer dental therapy, and these complications were correlated with a higher degree of myelosuppression [39]. Furthermore, the authors of another study reported that 10% of chemotherapy-treated cancer patients experienced febrile episodes due to dental sources [40]. In a more recent study, researchers reported that 3% of their total study population developed dental complications, with minimal treatment completed before the onset of cancer therapy [41].

**Figure 1:**
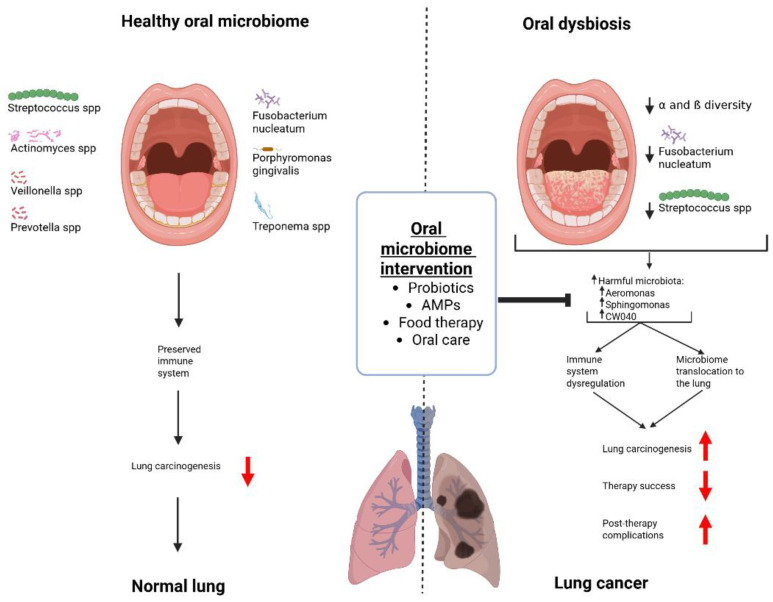
Summary of the correlation between the oral microbiome and lung cancer. Created in BioRender. Nagano, T. (2026) https://BioRender.com/a1a1i1a (accessed on 9 February 2026).

Lastly, it is important to note that overlaps exist between the oral dysbiosis observed in lung cancer and that reported in other forms of cancer. A relevant area for comparison is gastrointestinal cancers, such as gastric cancer or colon cancer. Indeed, the authors of multiple studies have highlighted these findings in both types of cancer. In gastric cancer patients, there are notable decreases in the commensal population of bacteria such as *Neisseria*, *Prevotella*, and *Haemophilus*; in colon cancer, in comparison, dysbiosis in oral pathogenic bacteria such as *Fusobacterium* or *Porphyromonas*, coupled with changes in those important in biofilm synthesis, such as *Streptococcus* and *Rothia*, are important markers for carcinogenesis. These findings underscore the common issue of oral dysbiosis, with common culprits across multiple types of cancer. As such, the importance of the oral microbiome must not be understated, and there is a necessity to include oral microbiota profiling to improve risk classification and outcome monitoring in the treatment of lung cancer. The points discussed in this section are summarized in Figure 1.

## 5. Oral Microbiome-Targeting Interventions

### 5.1. Probiotics

Although their wider mechanisms remain unclear, probiotics—live bacteria that provide health advantages when provided in sufficient amounts—are frequently found in food and supplements. Probiotics are believed to be useful as a method to rebalance the microbiome through multifaceted activities, including the colonization of host surfaces, antimicrobial metabolite production, and preventing pathogenic biofilm generation [42]. Importantly, several strains of probiotics have been reported to be efficient in improving lung cancer therapy outcomes or even assisting in supporting overall treatment efficacy. First, *Lactobacillus casei* is one of the most well-known probiotics currently available. It has been reported to have antibacterial effects in vitro against a broad spectrum of pathogens by producing metabolites that both promote an acidic condition and interfere with the bacterial cell membrane, potentially mitigating pneumonia-related complications and anticancer effects in A549 lung cancer cells through its antimicrobial effects [43,44]. The authors of a recent study highlighted how *Lactobacillus casei* strain Shirota, when combined with an oral nutrition supplement, could enhance the outcome of chemotherapy-treated lung cancer patients, underlining its potential [45].

Another potential strain is the probiotic *Clostridium butyricum* MIYAIRI 588 (CBM588), which has demonstrated efficacy as a therapeutic approach for gut dysbiosis [46]. *C. butyricum* has been reported to decrease the systemic inflammatory response, promote homeostatic maintenance, and lessen chemotherapy-induced diarrhea in lung cancer patients [47]. Findings from a 2024 report highlighted how CBM588 could be effective as a therapeutic adjuvant for periodontal treatment [46]. Building on the aforementioned report, the authors of another study demonstrated how ICI-treated NSCLC patients receiving MIYAIRI588 tended to exhibit prolonged survival compared to the untreated group [48]. Other probiotic strains reported to affect lung cancer therapy include BP-1 (which combines *Bifidobacterium lactis Bi-07*, *Lactobacillus acidophilus NCFM*, *Lactobacillus rhamnosus HN001*, and *Bifidobacterium lactis HN019*), *Pediococcus pentosaceus* FP3, *Lactobacillus salivarius* FP25/FP35, and *Lactobacillus rhamnosus* GG (LGG) [49,50]. In a pooled meta-analysis of various probiotics used in chemoradiotherapy-treated cancer patients, including lung cancer, it was found that, overall, the addition of probiotics could lead to a reduced occurrence of side effects, further promoting their potential [51].

Despite these advantages, there remain significant obstacles that must be overcome: overuse increases the risk of dysbiosis, and further research is needed to understand the mechanisms underlying probiotic-mediated anticancer effects. Furthermore, the latest results regarding probiotic use in lung cancer cast doubt over its efficacy; probiotic supplement use was not linked to substantial changes in survival rates in either the ICI atezolizumab or non-atezolizumab groups, based on the results of a recent study by Takada et al. [52]. These findings were consistent for both the general population and subgroups stratified by antibiotic exposure. To effectively use probiotics for cancer prevention and therapy, further research is needed to optimize strain-specific uses, dosage, and safety. At present, probiotics are deemed a viable adjunct in the treatment of respiratory and oral disorders because of their dual function in regulating immune responses and microbiomes; however, more evidence is needed to validate these findings.

### 5.2. Antimicrobial Peptides

A variety of small, naturally occurring peptide compounds known as antimicrobial peptides (AMPs) are essential components of the innate immune system [53]. Strong antibacterial, antiviral, antifungal, and anticancer capabilities are among the broad-spectrum biological activities displayed by these evolutionarily conserved chemicals. AMPs interact in concert with other antimicrobial components to construct a complex defensive system that preserves microbial balance and protects against potential pathogens. This effect is mediated by their distinct structural features, which enable them to interact with membrane proteins and ion channels to compromise the integrity of bacterial membranes, ultimately resulting in cytoplasmic leakage and cell death [54]. AMPs can be classified as either natural or synthetic. Depending on the species source, natural AMPs can be further classified as microbial, plant, animal, and so forth [55]. AMPs can also be categorized based on their primary functions, such as AMPs, anticancer peptides, and immunomodulatory peptides, given many of them have multiple roles in addition to direct antibacterial activity, including immunomodulation, anti-inflammation, anti-biofilm, anti-tumor, and promotion of wound healing [55].

As the potential complications of lung cancer therapies are closely related to the immune status of the patient, it is logical that changes in oral AMPs, either naturally or induced by outside intervention, could lead to modulation in therapeutic outcomes. First, β-defensins show broad-spectrum effectiveness against major periodontal pathogens such as *Aggregatibacter actinomycetemcomitans*, *Fusobacterium nucleatum*, and *Streptococcus pyogenes*, in addition to fungal species such as *Candida albicans* and non-albicans Candida [56]. In addition to their direct microbicidal actions through membrane rupture, β-defensins also aid in immune system regulation by encouraging T cell and dendritic cell chemotaxis, which links innate and adaptive immunity [56]. Several other important AMPs include LL-37, which performs distinct antibacterial processes by directly adhering to bacterial cell walls and disrupting biofilms and has considerable immunomodulatory capacity, and Histatins, which are potent antifungal defensive components that induce death in *Candida* [57,58].

Surprisingly, several AMPs exhibit promising antineoplastic qualities with specific activity against NSCLC. For example, *Lactococcus lactis* produces Nisin ZP, which inhibits the proliferation and migration of cancer cells while also inducing mitochondrial-mediated apoptosis and G0/G1 cell cycle arrest through ROS generation pathways, and has been formulated as a nasal spray [59]. Buforin IIb, through mitochondria-dependent apoptotic pathways, exhibits increased cytotoxicity against multiple in vitro and in vivo lung cancer models [60]. By disrupting calcium homeostasis, the AMP tilapia piscidin 4 (TP4), which is produced from Oreochromis niloticus, promotes an activator protein-1 (AP-1) protein called FosB [61]. Increased FosB may cause cancer cells to undergo apoptosis, as elevated levels of Fos and JUN proteins in conjunction with AP-1 have been reported in conditions in which cells undergo apoptosis. TP4 exerts two effects on lung cancer. First, TP4-mediated activation of FosB causes NSCLC cells’ cytoskeletal and membrane integrity to be disrupted. Second, protocadherin beta-13 (PCDHB13), which regulates and interferes with microtubule dynamics, is activated by FosB [62]. These mechanisms are consistent with clinical observations showing a negative correlation between the clinical course of NSCLC patients and both FosB and PCDHB13 expression. Lastly, while not directly related to lung cancer, Jiang et al. showed that administering chewing gum containing antimicrobial peptides such as GH12 to healthy volunteers increased the presence of *Actinobacteria*, *Leptotrichia*, and *Ciliophora*, all of which are important for oral homeostasis, while concurrently decreasing the abundance of potentially harmful species [63].

The above results demonstrate AMPs’ therapeutic promise in cancer, particularly for aggressive, metastatic NSCLC. These compounds are attractive prospects for development as adjuvant anticancer agents due to their complex modes of action, combining direct cancer-killing effects via membrane disruption, angiogenesis inhibition, and altering cancer cell metabolism, while also simultaneously modulating the microbiome. In future studies, researchers should focus on developing novel delivery methods, applying combinatorial strategies with existing treatments, and creating new AMP variants, in addition to expanding studies on currently available AMPs to realize their potential.

### 5.3. Food Therapy

The composition and function of oral microbial communities are greatly influenced by nutritional intake, suggesting a possible connection between food consumption and lung cancer, linked to the oral microbiome [64]. An increasing body of evidence indicates that some dietary elements may influence the oral microbiome to be protective rather than harmful to lung cancer [64]. Importantly, these effects are also linked with probiotics, whereby probiotic-rich foods, such as fermented dairy products, could help promote a more balanced oral microbiome and thereby provide a better environment to prevent cancer progression and optimize cancer treatment [50,65]. Concurrently, fruits and vegetables abundant in antioxidants (e.g., lemons or berries) have protective correlations and are recommended for maintaining the oral microbiome [66]. Another important element is dietary fiber, which supports oral and systemic health through its capacity to act as a substrate for microbial fermentation and maintain homeostasis. Through complex gut–oral axis signaling pathways, this microbial regulation results in decreased systemic inflammation [67]. Given the proven significance of inflammation in lung cancer, these anti-inflammatory properties seem especially pertinent for lung cancer prevention.

Importantly, as mentioned previously, a study combining oral nutrition supplementation with probiotics yielded a positive result in improving lung cancer chemotherapy outcomes [45]. The results of another study suggested that dietary differences could lead to increased or reduced adverse effects in lung cancer patients treated with ICIs [68]. In another study, these findings were corroborated, whereby dietary counseling could lead to significantly better treatment responses in lung cancer patients [69]. While these food-related interventions and nutritional aspects are deemed to be important in many aspects of the oral microbiome and its association with lung cancer, future studies are warranted to confirm the effects of nutritional intervention in altering oral dysbiosis and lung cancer therapeutic outcomes.

### 5.4. Oral Healthcare

The significant benefits of professional oral healthcare interventions have been demonstrated by a large body of evidence, especially for populations at risk of respiratory infections, such as lung cancer patients undergoing therapy [70]. When provided by dental professionals, comprehensive treatment that includes tongue, teeth, and denture cleanliness shows quantifiable preventive effects against pulmonary health issues. Professional oral hygiene interventions significantly reduce the incidence of aspiration pneumonia through various mechanisms, based on the results of systematic reviews [71]. Regular activities, including sufficient hydration and the chewing process, coupled with brushing and rinsing the teeth, are important in maintaining an adequate oral microbiome population [65]. Numerous studies have demonstrated a direct correlation between preoperative oral interventions, such as plaque care or oral rinses, and a decreased risk of postoperative pneumonia, highlighting the preventive utility of oral hygiene in surgical settings [72].

In conditions such as lung cancer, in which the immune system is compromised, and in those with restricted/reduced oral intake capability, notable changes in the oral microbiome are observed that predispose towards an increase in an opportunistic, pathogenic microbiome [73]. This state not only further weakens the immune system but could also contribute to its translocation to the lungs and contribute to both pulmonary infections and further progression of lung cancer. Importantly, oral hygiene practices such as brushing the teeth regularly can reduce the potential risk of the pathogenic microbiome (e.g., *Candida* or *Staphylococcus*) from translocating to the lung [74]

Periodontal therapy offers advantages when integrated into a pre-chemotherapy regimen, particularly before high-dose chemotherapy [41]. In addition to oral hygiene education, scaling and root planning procedures are used in periodontal therapy to physically disturb and debride germs and biofilm [75]. One of the most crucial and successful strategies for preventing bacterial recolonization and maintaining disease management is often oral hygiene education. Complete and differential blood counts may be necessary when performing these operations on immunocompromised patients in order to assess whether platelet transfusions are necessary before invasive periodontal treatment or whether antibiotics should be provided as an adjuvant. As such, ensuring that lung cancer patients receive appropriate oral hygiene care and periodontal therapy as required is important to maximizing lung cancer therapies. Currently available oral microbiome-modulating therapies are summarized in Table 2.

Through all the potential alterations in the oral microbiota as explained above, it becomes abundantly clear, however, that there is a need for large-scale studies involving a larger population to confirm the effects of oral microbiome modulation-based additional therapy on the success of lung cancer therapy, which should also be supported by appropriate mechanistic, preclinical studies. The currently available evidence is compounded by several limitations, such as the limited number of subjects and sampling sites, the lack of molecular mechanistic insights, and the lack of uniform evaluation/observation points and processes, making it difficult to generalize the findings to a wider population. Importantly, these therapies not only affect the oral microbiome but also the lung microbiome and even the central gut microbiome. Some therapies, such as probiotics or AMPs, have shown efficacy as additional treatment options in lung cancer, yet the number of studies addressing this particular aspect remains limited. As such, a greater number of large-scale, standardized studies are required to translate oral microbiome modulation into clinical use.

## 6. Conclusions

The oral microbiome is an often-overlooked but undoubtedly important aspect that can alter the trajectory and overall success of lung cancer therapy due to a multitude of underlying causes. Several intervention methods have been proposed to combat oral dysbiosis in recent years with varying degrees of success, including probiotics, antimicrobial peptides, food therapy, and oral care; however, more variations and larger-scale studies are needed to validate their usefulness in the setting of lung cancer treatment. As such, further studies are warranted to establish these interventions as an addition to current therapeutic options for lung cancer and to optimize their efficacy.

## Figures and Tables

**Table 1:** Key oral microbiome species.

Graph	Key Species	Physiological Roles	Typical Oral Site	Pathological Consequences
*Streptococcus* spp.	*S. mitis*, *S. sanguinis*, *S. mutans*	Early plaque colonizers, carbohydrate fermentation, acid production	Tongue, saliva, tooth surface, buccal mucosa, tongue dorsum, supragingival plaque	Major contributors to dental caries
*Actinomyces* spp.	*A. naeslundii*, *A. odontolyticus*	Early biofilm colonization, carbohydrate and protein metabolism	Supragingival plaque, subgingival plaque,	Cause of actinomycosis
*Veillonella* spp.	*V. atypica*, *V. parvula*	Lactate utilization, pH regulation, metabolic cooperation with *Streptococcus*	Tongue, saliva, subgingival plaque	Opportunistic pathogen in immune deficiency conditions
*Prevotella* spp.	*P. melaninogenica*, *P. intermedia*	Anaerobic protein degradation	Subgingival plaque	Inflammation associated with gingivitis/periodontitis
*Fusobacterium nucleatum*	*F. nucleatum*	Bridge species between early and late colonizers, strong adhesin activity	Subgingival plaque	Linked to colorectal and lung cancer
*Porphyromonas gingivalis*	*P. gingivalis*	Converting ethanol to acetaldehyde	Gingival crevice, subgingival plaque	Pathogen in periodontitis, immune evasion, protease production (gingipains)
*Treponema* spp.	*T. denticola*	Motile anaerobes, biofilm production that is synergistic with other bacteria	Gingival sulcus, subgingival plaque	Tissue-destructive proteases associated with advanced periodontal disease
*Candida* spp.	*C. albicans*	Fungal component, synergistic biofilm formation with bacteria	Tongue, mucosa	Key fungal pathogens in oral and lung infections
*Neisseria*	*N. subflava*, *N. mucosa*	Nitrate reduction, commensal colonization	Mucosal surfaces, saliva	
*Haemophilus*	*H. parainfluenzae*	Commensal; interacts with early biofilm	Buccal mucosa, saliva	Lung infection pathogens
*Rothia*	*R. dentocariosa*, *R. mucilaginosa*	Carbohydrate metabolism, commensal colonizer	Tongue dorsum, saliva	

**Table 2:** Currently known oral microbiome-targeting strategies.

Strategy/Treatment	Examples	Effects
Probiotics	*L. casei*	Together with paclitaxel, *L. casei* elicits anti-angiogenesis and pro-apoptotic effects on preclinical models Improved liver functions in patients undergoing therapy
	*Clostridium butyricum* MIYAIRI 588	Longer progression-free survival (9 months vs. 5 months without) and overall survival in NSCLC patients with chemotherapy
	BP-1	Reduce the incidence of chemotherapy-related nausea/vomiting (0% vs. 71%), constipation (2% vs. 63%), and diarrhea (7% vs. 42%)
	*Lactobacillus rhamnosus* GG	Together with paclitaxel, *Lactobacillus rhamnosus* GG prevents metastasis in preclinical models and decreases tumor growth in lung cancer models
Antimicrobial peptides	Nisin ZP	Exhibits anti-cancer effects in NSCLC cells
	Buforin IIb	Cytolytic activity against multiple cancer cells, including lung cancer cells
	Tilapia piscidin 4	Induces reactive oxygen species-mediated cytotoxicity in NSCLC cells
	Chewing gum containing AMPs	Direct rebalancing of the oral microbiome
Food therapy	Diet + Oral Nutrition Supplement containing additional carbohydrate, fat, and protein	Improves liver functions in patients undergoing therapy
	Dietary counseling	Increases the number of partial responders (60% vs. 48%) and stable disease (37% vs. 27%) patients after lung cancer therapy
	Vitamin K-rich or Keto-rich diet	Increases the progression-free survival of NSCLC patients
Oral care therapy	Preoperative/treatment plaque care and oral rinses	Decreases the incidence of post-operative pneumonia (7.5% vs. 9.2%) and fever

## Data Availability

The original contributions presented in this study are included in the article. Further inquiries can be directed to the corresponding author(s).

## Abbreviations

The following abbreviations are used in this manuscript:

NSCLC	Non-small cell lung cancer
TKI	Tyrosine kinase inhibitor
ICIs	Immune checkpoint inhibitors
EGFR	Epithelial growth factor receptor
PD-1	Programmed cell death protein 1
PD-L1	Programmed cell death ligand 1
CTLA-4	Cytotoxic T-lymphocyte-associated protein 4
AMP	Antimicrobial peptides
TP4	Tilapia piscidin 4
PDCHB13	Protocadherin beta-13
